# The viral vector vaccine VSV-GP boosts immune response upon repeated applications

**DOI:** 10.1186/1742-4690-9-S2-P301

**Published:** 2012-09-13

**Authors:** R Tober, Z Banki, A Ejaz, A Muik, L Egerer, D von Laer, J Kimpel

**Affiliations:** 1 Applied Virology and Gene Therapy Unit, Georg-Speyer-Haus, Frankfurt, Germany; 2 Innsbruck Medical University, Innsbruck, Austria

## Background

Vesicular stomatitis virus (VSV) is a potent candidate vaccine vector for various viral diseases (e.g. HIV, HCV, RSV). The biggest limitation of VSV, however, is its neurotoxicity, which limits application in humans. The second drawback is that VSV induces neutralizing antibodies rapidly and is thus ineffective as a vaccine vector upon repeated applications. Our group has recently shown that VSV pseudotyped with the glycoprotein (GP) of the lymphocytic choriomeningitis virus (LCMV), VSV-GP, is not neurotoxic. The aim of this project was to evaluate the potential of VSV-GP as a vaccine vector.

## Methods

For this purpose, we used Ovalbumin (OVA) as a model antigen and analyzed immunogenicity of GP-pseudotyped and wildtype VSV containing OVA (VSV-GP-OVA and VSV-OVA) in vitro and in vivo in mouse models.

## Results

We showed that both vectors infected murine bone marrow-derived dendritic cells (bmDCs) in vitro. These bmDCs were able to activate OVA specific CD8+ and CD4+ T cells. Immunization experiments in mice revealed that both VSV-OVA and VSV-GP-OVA induced functional OVA-specific cytotoxic T cells (CTLs) after a single immunization. In addition, with both viruses, mice generated antibodies against OVA. However, boosting with the same virus was only possible for the GP-pseudotyped virus but not for wild type VSV. The efficacy of repeated immunization with VSV-OVA was most likely limited by high levels of neutralizing antibodies, which we detected after the first immunization. In contrast, no neutralizing antibodies against VSV-GP were induced even after boosting.

## Conclusion

Taken together, we showed that the non-neurotoxic VSV-GP is able to induce specific T cell and B cell responses against the model antigen OVA to the same level as the wild type VSV vector. However, in contrast to wild type VSV, VSV-GP-OVA boosted the immune response upon repeated applications. Thus, VSV-GP is a promising novel vaccine vector.

